# Chicken Primordial Germ Cells Do Not Proliferate in Insulin-Lacking Media

**DOI:** 10.3390/ijms26073122

**Published:** 2025-03-28

**Authors:** Xin Liu, Jun Wu, Yixiu Peng, Hongwu Qian, Xiaoqian Lv, Fan Li, Kai Jin, Yingjie Niu, Jiuzhou Song, Wei Han, Guohong Chen, Bichun Li, Qisheng Zuo

**Affiliations:** 1 Key Laboratory of Animal Genetics, Breeding and Molecular Design of Jiangsu Province, College of Animal Science and Technology, Yangzhou University, Yangzhou 225009, China; 2 Joint International Research Laboratory of Agriculture and Agri-Product Safety of Ministry of Education of China, Yangzhou University, Yangzhou 225009, China; 3 Animal & Avian Sciences, University of Maryland, College Park, MA 20742, USA; 4 Poultry Institute, Chinese Academy of Agricultural Sciences Poultry Institute of Jiangsu, Yangzhou 225003, China; 5 College of Biotechnology, Jiangsu University of Science and Technology, Zhenjiang 212100, China

**Keywords:** primordial germ cells, insulin, RNA-seq, proliferation

## Abstract

Insulin is an important component of stem cell cultures; however, its role in the proliferation of avian primordial germ cells (PGCs) is unknown. The proliferation of PGCs in cultures varies and the growth factors and signaling pathways necessary to induce the proliferation of PGCs in chickens are unknown. Therefore, we conducted the present study to investigate the effect of insulin on the survival and proliferation of PGCs. In this study, we observed that under this culture system, PGCs proliferate in the presence of insulin, but do not proliferate in the absence of insulin. Furthermore, in insulin-lacking media, the expression of pluripotency genes, including *LIN28*, *NANOG*, *POUV*, and *SOX2,* was markedly decreased. Similarly, the expression of cell adhesion proteins *ZO-1*, *Occludin*, and *JAM-A* was significantly reduced. Elevated levels of ROS, GSSG, and MDA reduced the redox capacity of the cells and induced apoptosis. Subsequent transcriptome analyses revealed that insulin is one of the key factors in the proliferation of chicken PGCs through the regulation of downstream genes by PI3K/AKT, ECM–receptor interaction, Wnt, and P53 signaling, and that these downstream genes may be important for PGCs’ proliferation and survival.

## 1. Introduction

Avians are the animal model of choice for studies in developmental biology [1,2,3]. However, the process of embryonic development in avian species is different from other animals and zygotes are difficult to obtain; therefore, research on transgenic avian species has not made outstanding progress thus far. However, because of the various advantages of using avian species, the practical application of avian PGCs has attracted widespread attention, and the isolation and cultivation of PGCs has gained ground in the last decade. Therefore, the prospect of genetically modifying PGCs to prepare transgenic avian species and obtaining the desired production traits is very promising and will also bring inestimable benefits to society. In addition, due to national emphasis on the preservation of genomes in recent years, the use of avian species to study breed preservation technology is also a research trend.

After years of exploration, chicken PGCs have become one of few germ cells that can be cultured in vitro for a long period of time, but there are still problems, such as the inefficient establishment of the lineage and paucity of research on the function of various trophic factors in culture systems [4]. In 2015, Whyte et al. developed a culture system in which PGCs can self-renew in the absence of feeder cells using fibroblast growth factor 2 (FGF2), activin A, and insulin [5]. Determination of media conditions allows for the identification of key molecular pathways required for the self-renewal of PGCs.

Insulin is an important metabolic regulatory hormone secreted by pancreatic β-cells; its ability to induce cell proliferation has been demonstrated in a variety of cells, including hepatic cells [6], umbilical cord-derived mesenchymal stem cells (MSCs) [7], and chondrocytes [8]. Human embryonic stem cells (hESCs) die in the absence of insulin; therefore, insulin is considered to be a necessary media culturing E8-hESCs [9]. Stem cell proliferation and the maintenance of pluripotency are regulated by genes, such as *ECM*, *LIN28*, *SOX2*, and *NANOG* [10,11,12,13,14,15,16,17,18,19,20]. Cell–cell adhesion via the link between the ECM component fibronectin and integrin forms a complex with *ZO-1* in cells at the edge of migrating monolayers, regulating cells’ migration [21]. *ZO-1* regulates the assembly of tight junctions by integrating the actin cytoskeleton with transmembrane proteins such as Claudins and Occludin [22]. Remodeling of the ECM and changes in integrin signaling are associated with insulin [23,24]. In addition, the ECM can activate the mitochondria to participate in the oxidative stress response by releasing reactive oxygen species (ROS), which, in turn, oxidize proteins, lipids, and nucleic acids [25,26]. ROS play important roles as secondary messengers, involved in self-renewal, cell proliferation, and apoptosis in embryonic stem cells (ESCs) [27,28,29]. In addition, insulin inhibits apoptosis after cleavage and the dissociation of cysteine aspartate, and activates Wnt and the PI3K/AKT signaling pathway to promote cell survival [30,31,32]. However, whether lack of insulin affects the ECM, oxidative stress, signaling pathway, or the proliferation of PGCs has not been studied.

Although insulin is an important component in stem cell cultures, it is not clear how PGCs behave in the absence of insulin, whether insulin affects PGCs’ pluripotency and cell survival, or what molecular mechanisms are associated with insulin’s function. Here, we investigated the effect of non-insulin media on the proliferation, cell adhesion, apoptosis, and related signaling pathways of PGCs by RT–qPCR, CCK-8, and RNA sequencing. We are mainly interested in the mechanisms by which insulin promotes proliferation and signal transduction. This study provides basic data for establishing an ideal in vitro culture system for chicken embryo PGCs to promote the regulation of reproductive cell development and transgenic research.

## 2. Results

### 2.1. Culture and Characterization of Chicken Primordial Germ Cells

**Figure 1:**
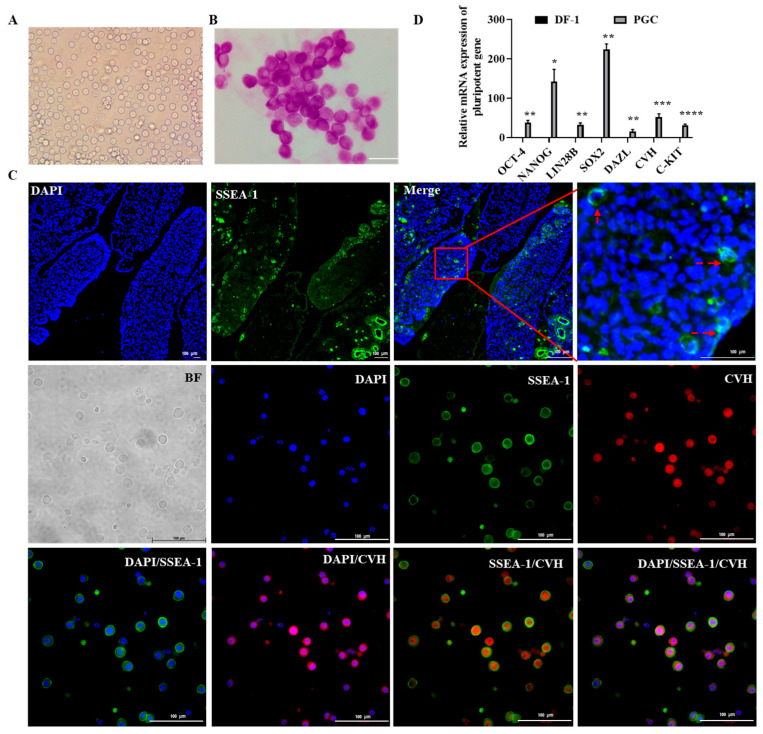
The culture and characterization of chicken primordial germ cells. (**A**) Morphological observation of PGCs cells after freezing and thawing; scale bar = 20 μm. (**B**) PAS staining diagram; bar = 10 μm. (**C**) Immunofluorescence of SSEA-1/CVH in PGCs in the gonads and PGCs cultured in vitro; bar = 100 μm. Red squares is a partial enlargement of a specific area in the ‘Merge’ image, used to display details more clearly, red arrow indicates PGCs (**D**) Expression of pluripotency and germline marker genes; *n* = 3, * *p* < 0.05, ** *p* < 0.01, *** *p* < 0.001, and **** *p* < 0.0001.

After 60 days of purification and culture, a morphologically uniform cell line was obtained (Figure S1) [5]. Morphological observation after freezing and thawing showed that the cultured cells grew in suspension and appeared circular and separately dispersed, which was consistent with the basic morphology of PGCs (Figure 1A) [33]. To further demonstrate whether the cultured cells had the biological properties of PGCs, this experiment systematically identified the cultured cells. The results showed that these cells presented PAS positivity and SSEA-1/CVH positivity, indicating that a large number of lipid droplets had attached to the cells’ surface, as well as the cells’ stemness (Figure 1B,C), consistent with the positive expression of SSEA-1 in PGCs in the gonads. PGCs are pluripotent, while somatic cells are not [33]. Therefore, RT–qPCR was then conducted and the results showed that the totipotency genes *NANOG*, *Oct4*, *SOX2* and germline marker genes *DAZL*, *CVH*, and *C-kit* were significantly more abundant in PGCs than in somatic cells (DF-1) (Figure 1D). All these results indicated that PGCs were successfully cultured in this study.

### 2.2. Insulin Is Indispensable for the Long-Term In Vitro Culture of PGCs

**Figure 2:**
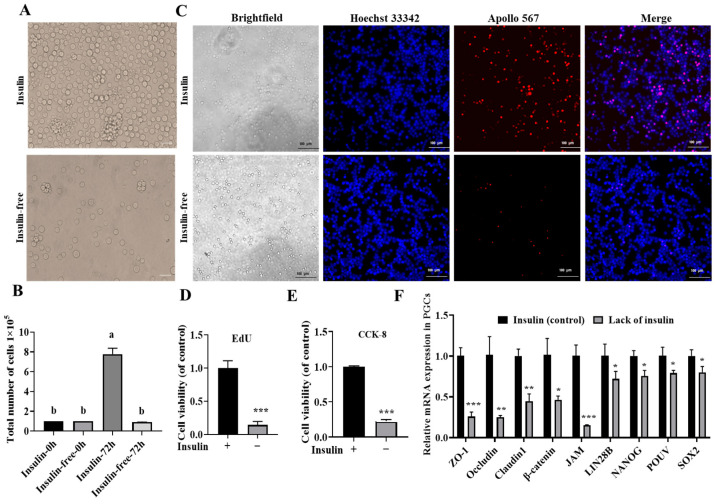
Insulin is indispensable for the long-term in vitro culture of PGCs. (**A**) Morphological observation of two groups of cultured PGCs; bar = 20 μm. (**B**) Cell counts; different superscripts represent significant differences. (**C**,**D**) EdU experimental fluorescence plots and statistical results. (**E**) The statistical results of the CCK-8 assay. (**F**) Expression of cell adhesion and pluripotency genes; * *p* < 0.05, ** *p* < 0.01, *** *p* < 0.001.

To investigate the effect of insulin on the proliferation of PGCs, B-27™ plus supplement (50×) was replaced with B-27™ minus insulin and the cells were cultured for 72 h and observed under a microscope. The results showed (Figure 2A) that there were significant proliferative changes in the insulin group and cell counts revealed that the cells in the non-insulin group did not proliferate (Figure 2B). Further proliferation assays using the CCK-8/EdU method showed (Figure 2C–E) that the efficiency of proliferation was highly significantly reduced in the non-insulin group compared to the control group (*p* < 0.001).

The proliferation of chicken embryonic intestinal cells is regulated by cell adhesion [34]. To further investigate whether the lack of insulin affected the cells’ adhesion, this study used qRT–PCR to detect the expression of cell adhesion-related genes. As shown in Figure 2F, the expression levels of *ZO-1*, *Occludin*, *Claudin1*, *JAM*, and *β-catenin* mRNA were significantly lower in the non-insulin group than in the insulin group. These results showed that non-insulin media reduce the adhesion capacity of PGCs and thus affect cell proliferation.

### 2.3. Non-Insulin Media Leads to Decreased Expression of Pluripotency Genes

To investigate the effect of non-insulin media on the pluripotency of PGCs, we first examined the expression of pluripotency genes, including *POUV*, *SOX2*, *NANOG*, and *LIN28B*. Figure 2F shows that the expression levels of *POUV*, *SOX2*, *NANOG*, and *LIN28B* were reduced in media without insulin, indicating that non-insulin media reduced the expression of pluripotency genes in PGCs.

### 2.4. Non-Insulin Media Reduces Redox Capacity in PGCs

**Figure 3:**
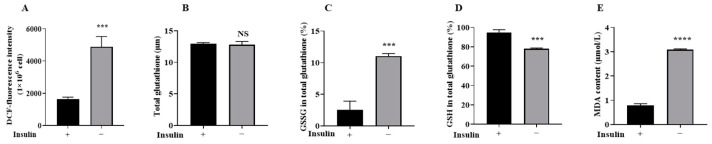
Non-insulin media reduces redox capacity in PGCs. (**A**) A statistical graph of the change in ROS content of the two groups. (**B**–**D**) Statistical analyses of changes in the intracellular GSH/GSSH ratio. (**E**) The fffect of the non-insulin medium on MDA levels; *n* = 3, *** *p* < 0.001, **** *p* < 0.0001.

To investigate the effect of non-insulin media on the redox capacity of PGCs, the content of the secondary messenger ROS was examined and it was found (Figure 3A) that the ROS content significantly increased, indicating that the cellular redox capacity of PGCs was reduced. ROS can cause damage to cells through lipid peroxidation [35]; moreover, MDA and GSSG were detected and the results showed (Figure 3B–E) a significant increase in MDA and GSSG content and a decrease in glutathione (GSH) content (*p* < 0.05). These results suggest that the cellular redox capacity of non-insulin PGCs was reduced.

### 2.5. Non-Insulin Media Can Induce Apoptosis in PGCs

**Figure 4:**
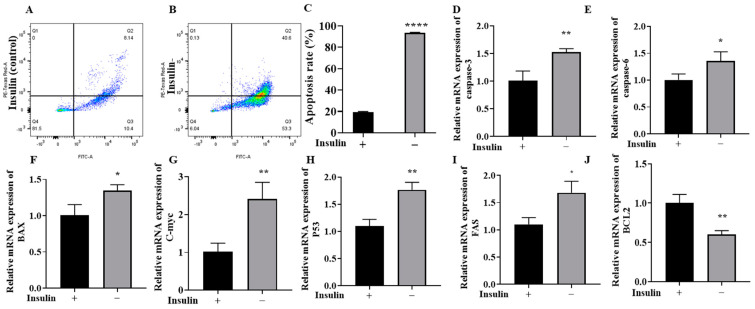
Non-insulin media can induce apoptosis in PGCs. (**A**–**C**) Apoptosis measured by flow cytometry and graphs of the statistics. (**D**–**J**) The effect of non-insulin media on apoptosis genes in the PGCs. *n* = 3, * *p* < 0.05, ** *p* < 0.01, and **** *p* < 0.0001.

ROS-mediated lipid peroxidation and its products can trigger apoptosis [36]. Therefore, the effect of lack of insulin on apoptosis in PGCs was explored next (Figure 4A–C). We first found a significant increase in apoptosis in the non-insulin group by flow cytometry with FITC/PI labeling (*p* < 0.05). Next, qRT–PCR was used to detect the expression of apoptosis-related genes. The results showed that the expression levels of the apoptosis-related marker genes *caspase-3*, *caspase-6*, *C-myc*, *BAX*, *P53*, and *FAS* were significantly upregulated in the PGCs after treatment with non-insulin media (Figure 4D–I). In contrast, the expression of the antiapoptotic marker *BCL-2* was significantly downregulated (Figure 4J) (*p* < 0.05). Downregulation of *BCL-2* and upregulation of BAX are important markers of apoptosis in cells [37], indicating that the non-insulin media induced apoptosis in the PGCs.

### 2.6. RNA-Seq Assessment of the Quality of the Data

**Figure 5:**
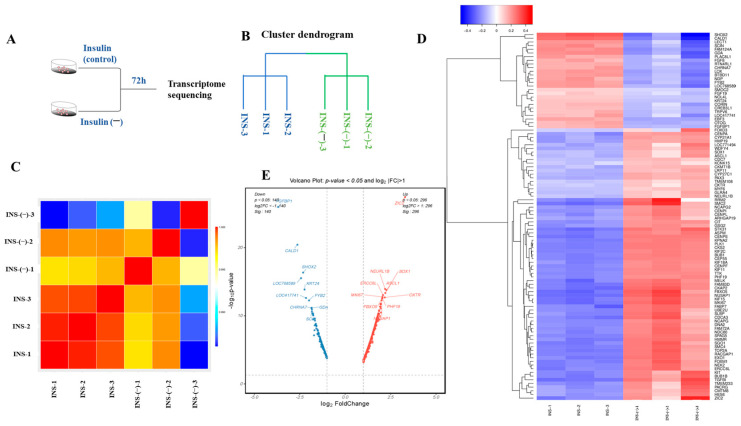
RNA-seq assessment of the quality of the data. (**A**) Sample source. (**B**) An example of a dendrogram comparing the non-insulin and insulin-treated groups. (**C**) An example of a correlation analysis where red means a strong correlation and blue means a weak correlation. (**D**) A statistical map of differential genes. The red columns represent upregulated genes and the blue columns represent downregulated genes. (**E**) A volcano map of differentially expressed genes between the non-insulin and insulin-treated samples. The red dots represent significantly upregulated genes; the blue dots represent significantly downregulated genes.

To investigate the differential genes and signaling pathways between the non-insulin and insulin groups (Figure 5A), this study used the Illumina TruSeqTM RNA sample preKit to construct six libraries and obtain gene expression profiles of the two groups. According to the results of the sample clustering and correlation analysis (Figure 5B,C), duplicate samples were tightly clustered and different samples were relatively dispersed, indicating that the test samples exhibited high repeatability and low dispersion. The results indicated that the sequencing data were reliable and could be used for subsequent analyses of differentially expressed genes and signaling pathway enrichment. DEGs between the different groups were screened based the threshold *p* < 0.05 and |Log2FC| > 1. The results showed (Figure 5D,E) that there were 436 DEGs between the non-insulin and insulin groups, of which 296 genes were upregulated and 140 genes were downregulated (Table S1).

### 2.7. Enrichment Analysis of DEGs

**Figure 6:**
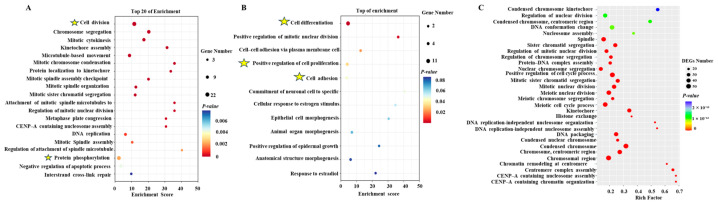
The GO enrichment analyses of the DEGs between the non-insulin and insulin-treated samples. (**A**) The GO analysis of all differentially expressed genes. Biological processes were mainly enriched in cell division and protein phosphorylation. (**B**) The GO analysis of all downregulated genes. (**C**) The GO analysis of all upregulated genes. Cell differentiation, cell adhesion, etc., are marked with an asterisk.

**Figure 7:**
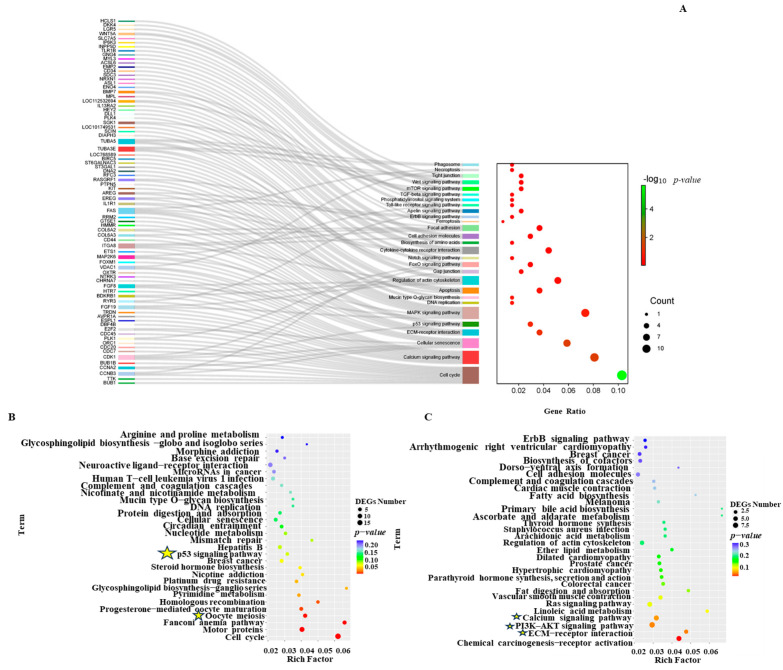
KEGG enrichment analyses of the DEGs between the non-insulin and insulin-treated samples. (**A**) The KEGG pathway enrichment analysis of all genes. (**B**) The KEGG pathway enrichment analysis of all upregulated genes. (**C**) The KEGG pathway enrichment analysis of all downregulated genes. Pathways of interest for the subsequent heatmap analyses are marked with an asterisk.

**Figure 8:**
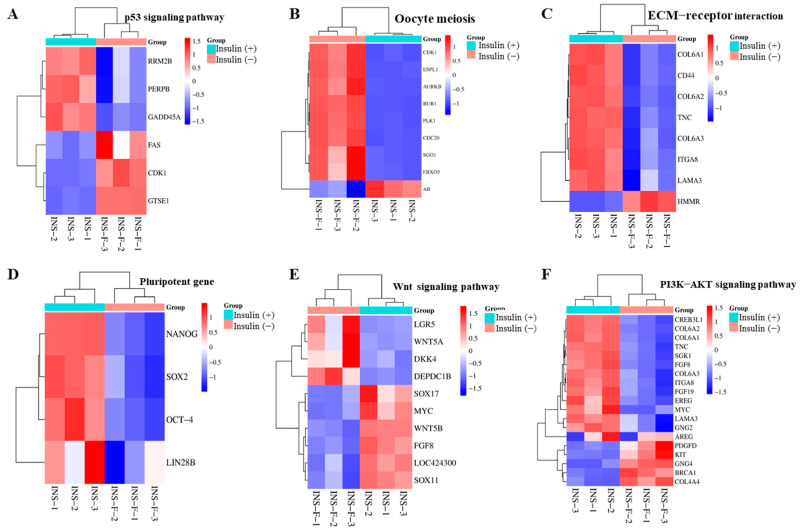
Heatmaps showing the clustering of signaling pathway genes. (**A**): p53 signaling pathway related gene expression heatmap; (**B**): Heat map of gene expression related to meiosis in oocytes; (**C**): Heat map of gene expression related to ECM−receptor interaction; (**D**): Heat map of Pluripotent gene expression; (**E**): Wnt signaling pathway related gene expression heatmap; (**F**): PI3K−AKT signaling pathway related gene expression heatmap.

GO analysis revealed that in all the differential genes, biological processes were mainly enriched in the pathways of cell division and protein phosphorylation (Figure 6A). Among the downregulated genes, GO analysis revealed them to be enriched in the pathways of cell differentiation, positive regulation of cell differentiation by proliferation, and cell adhesion (Figure 6B). Among the upregulated genes, GO analysis found them to be mainly enriched in the nucleosome assembly and sister chromatid segregation pathways (Figure 6C). To further analyze which signaling pathways were involved in regulating insulin to promote the proliferation of chicken PGCs, a KEGG analysis of the differentially expressed genes was performed. All the DEGs were mainly enriched in the calcium signaling pathway, the Wnt signaling pathway, cell adhesion, the mTOR signaling pathway, the FoxO signaling pathway, and ferroptosis (Figure 7A). All upregulated genes were enriched in the oocyte meiosis, progesterone-mediated oocyte maturation, steroid hormone biosynthesis (CYP1B1 and CYP21A1), P53 signaling pathway, and ferroptosis pathways (Figure 7B). All downregulated genes were enriched in the calcium signaling pathway, the Ras signaling pathway, ECM–receptor interactions, cell adhesion, and the PI3K/AKT signaling pathway (EREG and AREG) (Figure 7C). These results suggest that these signaling pathways play important roles in insulin-induced proliferation of chicken PGCs. Further heatmap (Figure 8A–F) analyses of the genes’ enrichment in the signaling pathways and comparison to previous experimental results again verify that lack of insulin reduces cells’ adhesion and pluripotency and promotes apoptosis in PGCs.

### 2.8. Non-Insulin Media Affect the PI3K/AKT and Wnt Signaling Pathways in PGCs

**Figure 9:**
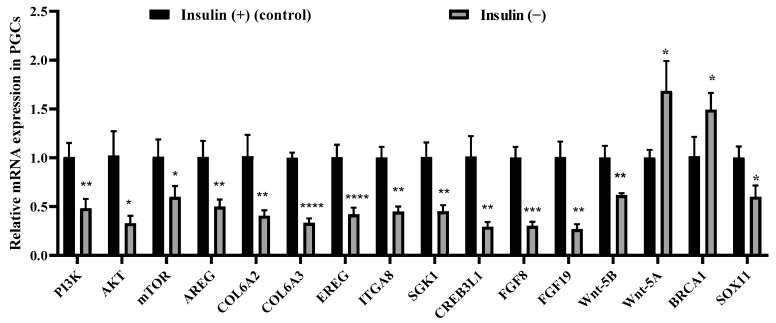
Non-insulin media inhibit the PI3K/AKT signaling pathway in PGCs. The effect of insulin media on the PI3K/AKT and Wnt signaling pathways genes detected by qRT–PCR; *n* = 3, * *p* < 0.05, ** *p* < 0.01, *** *p* < 0.001, and **** *p* < 0.0001.

Based on the sequencing results described above, we verified the expression of genes related to the PI3K/AKT and Wnt signaling pathways by qRT–PCR (Figure 9A) and compared the fold change of qRT–PCR with the results of the RNA-seq (Figure S2) expression analysis, and the results were consistent. The qRT–PCR results showed that *PI3K*, *AKT*, *mTOR*, *AREG*, *COL6A2*, *COL6A3*, *EREG*, *ITGA8*, *SGK1*, *CREB3L1*, *FGF8*, *FGF19*, *Wnt-5B*, and *SOX11* expression levels were significantly decreased, while the *Wnt-5A* and *BRCA1* expression levels were significantly upregulated. These results suggested that the PI3K/AKT and Wnt signaling pathways in the PGCs were inhibited by non-insulin media, thereby affecting proliferation.

## 3. Discussion

The proliferation of PGCs is an extremely complex process regulated by multiple factors, such as cytokines, key genes, and signaling pathways [38]. The proliferation of PGCs in cultures is different in that an appropriate growth factor must be added to the medium [39,40]. This experiment found that PGCs in non-insulin media do not proliferate and that decreased pluripotency, cell adhesion, and decreased redox capacity lead to apoptosis. The results from the transcriptomic data analysis suggest that non-insulin media are downregulated the PI3K/AKT and Wnt pathways. The results of this study clarify the role of insulin in the proliferation of chicken PGCs and lays a foundation for further studies of its mechanism.

Insulin has been used to improve stem cell cultures [9], and promotes hESC proliferation and cell adhesion [30]. E-cadherin is a classic cadherin protein [41]. E-cadherin and α/β-catenin are activated during the proliferation of PGCs and the inhibition of E-cadherin leads to cell death and affects the development of PGCs [16,42,43]. A reduction or loss in ZO-1 can lead to slowed cell proliferation and embryonic lethality [44,45]. JAM-A deficiency or overexpression can regulate 293T human embryonic kidney epithelial cell adhesion [46]. The results of this experiment indicate that JAM-A and ZO-1 decreased in the non-insulin group and ECM–receptor interactions were downregulated in the transcriptome, suggesting that the non-insulin media suppresses the expression of adhesion-related factors and, thus, the proliferation of PGCs.

Under physiological conditions, insulin binds to insulin receptor substrate 1 (IRS-1); IRS-1 promotes IGF-1 signaling [47], which, in turn, promotes PI3K, then AKT and downstream targets, such as mTOR, GSK3β, and FOXO [48,49,50]. In human embryonic stem cells, the pluripotency marker gene *Lin28* and related protein are decreased after knockdown of IGF-1. *Lin28* is essential for the maintenance of chicken PGCs and can regulate the cells’ proliferation in vitro [18]. It has been shown that activation of the IGF1R–/PI3K/AKT pathway increases the expression of cancer stemness markers NANOG and SOX2 [51]. In chicken PGCs, NANOG is responsible for regulating stem cells’ pluripotency during self-renewal [10,13], forming a transcriptional network with OCT4 and SOX2 [52,53]. In this study, these genes were all downregulated, which indicates a gradual loss of pluripotency-related features [52,53]. The results of this study show that in non-insulin media, self-renewal was interrupted and the pluripotency of the PGCs decreased, which are consistent with other research findings.

Insulin induces downregulation in the IGF-1R signaling pathway in adipose-derived stem cells (ADSCs), which, in turn, results in oxidative stress, leading to a significant increase in apoptosis [54]. Redox homeostasis is essential for cellular function [55] and for increased levels of reactive oxygen species (ROS) in ESCs under oxidative stress [27]. When the concentration of ROS increases, it can cause damage to DNA and decrease glutathione (GSH) content [56,57]. The content in the oxidative stress biomarker malondialdehyde (MDA) increases after sustaining damage to cell membranes [25,26,58,59]. Disruption of the ROS/GSH balance leads to inhibited proliferation of cells and even induces cell death [60]. In Type 2 diabetic rats, insulin can reduce MDA levels in the pancreas [61]. Previous research findings indicate that insulin significantly reduces the production of ROS in human plasma [62,63] and our results are consistent with these previous studies.

ROS-mediated lipid peroxidation and its products can trigger apoptosis [36] and antioxidants increase the number of porcine PGCs in vitro by inhibiting apoptosis [64]. In addition, activation of the p53 pathway can lead to reduced proliferation of PGCs [65]. Apoptosis is mainly regulated by the *BCL-2* protein family and the ratio of *BCL-2*/Bax proteins determines cell survival and death [66,67,68,69]. The normal development of germ cells is also highly dependent on the balanced regulation of mitochondrial apoptosis by *BCL-2*-family proteins and abnormal apoptotic genes impair germ cells’ differentiation [70]. A previous study suggests that the absence of insulin can lead to increased caspase-3 activity in rat hepatocytes, which may induce apoptosis [71]. Insulin significantly prevents apoptosis in rat liver cells by reducing the levels of mitochondrial pro-apoptotic Bax and cytoplasmic cytochrome C [63]. Our results are consistent with previous studies.

Insulin plays a crucial role in the formation of chicken PGCs [5]; however, the mechanism by which it regulates the proliferation of chicken PGCs is unclear. In this study, transcriptome sequencing revealed that the Wnt signaling pathway, oocyte meiosis, progesterone-mediated oocyte maturation, steroid hormone biosynthesis, and the P53 signaling pathway were involved (Figure 7A). Strict regulation of the Wnt signaling pathway is necessary for the proper development of PGCs [72,73,74]. Activation of the P53/BAX signaling pathway can induce apoptosis in hESCs [75], which is also consistent with results from our previous test. It has been shown that insulin is virtually independent of meiosis in goldfish and zebrafish oocytes [76,77] and has also been shown to induce steroidogenesis in vertebrate follicles and to induce maturation in oocytes [78]. Our transcriptomic data suggest that non-insulin media affect oocytes’ meiosis, which may provide some reference for the study of oocyte maturation; however, the exact mechanism deserves further investigation. The cortisol-related biomarker CYP1B1 [79] is the basic enzyme in the production of estrogen metabolites [80]. CYP21A1 is involved in the steroid hormone biosynthesis pathway and the overexpression of CYP21A1 promotes cells’ proliferation and inhibits apoptosis [81]. We infer that CYP1B1 and CYP21A1 do not promote the synthesis of steroid hormones at the gene level, but may promote the synthesis of steroid hormone proteins. The specific reasons need to be studied further. Downregulation in the calcium signaling pathway has also been found, with previous studies showing that insulin reduces calcium ions in mice pancreata, stimulates the activation of integrin in human embryonic stem cells, and promotes cell–matrix and cell–cell adhesion [30,82]. Compared with mammals, avian species have relatively high blood glucose levels. In obese chickens, the regulation of adipogenesis and fat deposition mediated by insulin signaling differs from that in obese mammals. Therefore, caution should be exercised when applying research findings from mammals to poultry studies [83]. The transcriptome results presented in this paper provide a theoretical basis for subsequent research on the mechanism of insulin in poultry.

Insulin activates the PI3K/AKT signaling pathway by binding to its receptor [84]. In human and mouse embryonic stem cells, the PI3K/AKT pathway is important for maintaining pluripotency, proliferation, and survival [85,86,87,88,89,90,91,92]. AREG activates the phosphoinositide 3-kinases (PI3K)/AKT/mammalian target of the rapamycin (mTOR) pathway and promotes the cycling of hematopoietic stem cells (HSCs) [93]. Insulin also can activate the Wnt signaling pathway, notably enhancing the expression of Wnt-5B in embryonic mesenchymal cells [94,95]. The functions of the Wnt and FGF signaling pathways in growing embryos are associated with various developmental processes, including cell proliferation, differentiation, migration, and apoptosis [96]. Notably, overexpression of FGF-8 can promote self-renewal in chicken PGCs [97]. Our results indicate that insulin can activate the PI3K/AKT and Wnt-5B/FGF8 signaling pathways in PGCs, consistent with previous studies.

## 4. Conclusions

To the best of our knowledge, this study is the first to systematically investigate the effect of insulin on the proliferation of PGCs and to identify the underlying regulatory mechanisms. Our results indicate that PGCs do not proliferate in non-insulin media and that decreased cell adhesion, cell pluripotency, and redox capacity lead to apoptosis and the downregulation of signaling pathways such as PI3K/AKT. This study contributes to a better understanding of the molecular regulation of insulin in PGCs and provides a theoretical reference for the optimization of the culture system, as well as studies signaling pathways in PGCs.

## 5. Materials and Methods

### 5.1. PGC Culture Conditions

The left gonads of 4.5-day-old Rugao yellow chicken embryos (30 pieces, repeated three times) were removed and placed in 200 μL of sterile BSA solution (Bioss, Beijing, China), crushed with a pestle, and digested with accutase enzyme (Lianke, China). After digestion for 10 min, an equal volume of the complete PGC medium (Table 1) was added, the supernatant was discarded by centrifugation, and the 48-well cultures were resuspended and incubated at 37 °C in a 5% CO_2_ incubator (Thermo Fisher Scientific Inc., Shanghai, China). The cultures were observed daily and the medium was changed every 3 days. Following a period of 60 days of purification and cultivation, all PGCs in the culture dish were collected. The efficiency of cell line establishment was 30% (9/30, 9/30, 9/30) and cells were frozen using serum-free cryopreservative (Ncmbio, Changzhou, China). The cells were thawed and subsequently used for downstream experimentation, encompassing all the mentioned procedures, such as PAS staining, drug treatment, cell counting, EdU proliferation assay, RNA extraction and RT–qPCR, ROS, GSH/GSSG, MDA, Western blotting, and transcriptome sequencing.

### 5.2. Periodic Acid–Schiff Stain

This assay used cells fixed in 4% paraformaldehyde, rinsed in distilled water, placed in Reagent A for 8 min, rinsed, and stained with Reagent B Schiff for 15 min. The membrane was rinsed and sealed, and observed under an Olympus BX41 microscope. The specific steps were conducted as specified by the periodic acid–Schiff (PAS) staining kit (Solarbio, Beijing, China).

### 5.3. Immunohistochemistry

The gonads were fixed with 4% paraformaldehyde for 24 h, after which they were trimmed and passed through alcohol and xylene for dehydration and transparency and dipped in wax for embedding. The trimmed wax blocks were frozen at −20 °C for 30 min and then sliced. When the tissue temperature was −20 °C, thinner continuous slices could be cut. The slices were submerged in warm water at 40 °C, the specimens were fixed with adhesion microscope slides (CITOTEST, Nantong, China), and the slides were placed in an oven at 37 °C for 12 h, after which they were stored at room temperature. The PGCs were fixed on adhesion microscope slides with 4% paraformaldehyde, infiltrated with Triton X-100, and washed three times with PBST. The cells and gonads were sealed with normal goat serum for 2 h and 6 h (Solarbio, Beijing, China), respectively. After incubation with primary antibodies against SSEA-1 (Abcam, Shanghai, China) or DDX4/MVH (Abcam, Shanghai, China) at 4 °C overnight, the membrane was washed three times with PBST and incubated with the corresponding secondary antibodies (Alexa-Fluor 488 (Abcam, China) and goat anti-rabbit IgG H&L/Cy3 (Bioss, Beijing, China)) for 2 h at room temperature. The sections were rinsed three times with PBST, titrated with the mounting medium and an antifading agent (DAPI) (Beyotime, Shanghai, China), sealed and visualized using a Leica microscope (Leica Microsystems Inc., Wetzlar, Germany).

### 5.4. Experimental Design

PGCs in the logarithmic growth phase were inoculated into 24-well plates (1 × 105) and divided into two groups. The controls received B-27™ plus supplement (50×) (Thermo Fisher Scientific Inc., Shanghai, China) and the experimental treatment received B-27™ minus insulin (Thermo Fisher Scientific Inc., Shanghai, China) for 72 h for subsequent analyses. The only difference between the two groups was that the experimental group did not have insulin.

### 5.5. Cell Counting Kit-8 (CCK-8) Assay

For this assay, 10 μL of CCK-8 reagent was added to 100 μL of culture medium. Three parallel wells were set up for the experimental, control, and blank groups and PBS was added to the periphery of the unused wells to prevent evaporation of the liquid. The samples were incubated at 37 °C for 2 h in a 5% CO_2_ incubator, after which absorbance was measured at 450 nm (SPARK, Grödig, Austria). The cell counting kit (CCK-8) (Dojindo, Beijing, China) was used in these specific steps.

### 5.6. EdU Proliferation Assay

In total, 1 × 10^5^ cells per well were taken and cultured in EdU medium at a concentration of 50 μm (this concentration was obtained through preliminary experimentation) for 2 h. The culture solution was discarded for washing and the cells were fixed on slides with 4% paraformaldehyde, washed, permeabilized with Triton X-100, and stained with Apollo staining. The sections were incubated with a mounting medium and an antifading solution (DAPI) and sealed and observed with a Leica microscope. The procedure was carried out according to the manufacturer’s instructions from the Cell-Light EdU Apollo567 In Vitro Kit (Ribobio, Guangzhou, China).

### 5.7. RNA Extraction and Real-Time Quantitative PCR (RT–qPCR)

Total RNA was isolated using 1 mL of TRIzol reagent (Thermo Fisher Scientific Inc., Shanghai, China), according to the manufacturer’s protocol. A nanodropper was used to measure the concentration of RNA (NanoDrop 1000, Shanghai, China). Reverse transcription to cDNA was carried out using FastKing gDNA Dispelling RT SuperMix (TIANGEN, Beijing, China). RT–qPCR amplification was performed on a CFX Connect™ Real-Time System (Bio-Rad, Hercules, CA, USA) using ChamQ Universal SYBR qPCR Master Mix (Vazyme, Nanjing, China). The reaction conditions were 95 °C for 5 min followed by 40 cycles of 95 °C for 10 s and 60 °C for 30 s. After PCR amplification, a melting curve was obtained, namely, 95 °C for 15 s, 60 °C for 1 min, and 95 °C for 15 s, to verify the primers’ specificity. The relative expression of genes was calculated using the 2^−ΔΔCt^ method after normalization to the mRNA expression of the housekeeping gene β-actin. The primers were synthesized by Beijing Tsingke Biotech; their sequences are shown in Table S1.

### 5.8. Detection of Apoptosis by the FITC/PI Double-Staining Method

After centrifugation at 1000 rpm for 5 min, the cells were collected, washed twice with precooled PBS, and 5 × 10^5^ cells were collected. Next, 100 μL of 1× binding buffer, 5 μL of Annexin V-FITC, and 10 μL of PI staining solution were added. The mixture was incubated for 15 min at room temperature in the dark. After that, 400 μL of 1× binding buffer was added and the mixture was assayed by flow cytometry. For the specific steps, please refer to the Annexin V-FITC/PI Apoptosis Detection Kit (Yeasen Biotechnology, Shanghai, China). We distinguished between living cells, dead cells, early apoptotic cells, and apoptotic cells. The relative proportion of early apoptotic cells (Q3) and apoptotic cells (Q2) was combined as the target of our comparison [98].

### 5.9. Reactive Oxygen Species Assay

Cells were collected by centrifugation, added to 10 μM DCFH-DA, incubated at 37 °C for 2 h, and mixed every 10 min. The cells were washed with a serum-free cell culture solution to remove DCFH-DA that did not enter the cells, which was detected using a microplate reader. For the specific steps, please refer to the Reactive Oxygen Species (ROS) Assay Kit (Yeasen Biotechnology, Shanghai, China).

### 5.10. Assay for GSH/GSSG/MDA

The content of reduced glutathione (GSH) and oxidized glutathione disulfide (GSSG) was assayed with a GSH and GSSG assay kit according to the manufacturer’s instructions (Beyotime, Shanghai, China). GSH content of the test samples was calculated as the total glutathione (GSSG) × 2. Malondialdehyde (MDA) content was measured using a lipid peroxidation MDA assay kit according to the manufacturer’s instructions (KeyGEN BioTECH, Nanjing, China). After centrifugation at 12,000× *g* at 4 °C for 10 min, the supernatant was harvested and assayed with a microplate reader.

### 5.11. RNA Extraction, Library Preparation, and Illumina HiSeq Sequencing

Total RNA was extracted from the PGCs using TRIzol^®®^ reagent according to the manufacturer’s instructions (Invitrogen, Waltham, MA, USA), and genomic DNA were removed using DNase I (TaKaRa, Kusatsu, Shiga). Then, the quality of RNA was determined using a 2100 bioanalyser (Agilent, Santa Clara, CA, USA) and quantified using the ND-2000 spectrophotometer (NanoDrop Technologies, Wilmington, DE, USA). RNA-seq transcriptome libraries were prepared following instructions from the TruSeqTM RNA Sample Preparation Kit from Illumina (San Diego, CA, USA). The experiments were performed by the Shanghai Biozeron Biotechnology Co., Ltd. (Shanghai, China).

### 5.12. Differential Expression Analysis and Functional Enrichment

To identify differential expression genes (DEGs) between the two different samples, the expression level of each gene was calculated using the fragments per kilobase of exon per million mapped reads (FRKM) method. The R statistical package edgeR (https://www.bioconductor.org/packages/release/bioc/html/edgeR.html) was used for differential expression analysis (accessed on 27 September 2024). DEGs between two samples were selected using the following criteria: the logarithm of the fold change is greater than 2 and a false discovery rate (FDR) less than 0.05. To understand the functions of the differentially expressed genes, GO functional enrichment and KEGG pathway analyses were performed via https://bioinformatics.com.cn/ (accessed on 27 September 2024).

### 5.13. Statistical Analysis

Data are presented as the mean ± SEM. Statistical analyses were performed using SPSS (version 25.0, USA) and GraphPad Prism (GraphPad Software, version 10, USA). Histograms were drawn and statistical significance was set to *p* < 0.05.

## Figures and Tables

**Table 1:** Cell culture medium.

DMEM Basal Medium			
Composition	Source	Concentration	Volume
DMEM	Meilunbio-PWL037, Dalian, China	75%	37.5 mL
Ultra-filtered water	Sigma-W3500, Shanghai, China	24%	12 mL
CaCl_2_·2H_2_O	Sigma-C7902, Shanghai, China	0.15 mM	0.5 mL
DMEM basal medium	Discard 3.368ml		46.632 mL
B-27^TM^ supplement (control)	Gibco-17504044, Shanghai, China	1×	1 mL
B-27^TM^ supplement, minus insulin (experimental group)	Gibco-A1895601, Shanghai, China	1×	1 mL
GlutaMax	Gibco-35050061, Shanghai, China	2 mM	0.5 mL
MEM NEAA	Gibco-11140050, Shanghai, China	1×	0.5 mL
2-Mercaptoethanol	Gibco-21985023, Shanghai, China	0.1 mM	91 µL
Chicken serum	Gibco-16110082, Shanghai, China	0.2%	100 µL
EmbryoMax nucleosides	Sigma-ES-008-D, Shanghai, China	1×	0.5 mL
Sodium pyruvate	Gibco-11360070, Shanghai, China	1.2 mM	0.6 mL
Ovalbumln	Sigma-A5503, Shanghai, China	0.2%	0.1 g
Sodium heparin	MCE-HY-17567A, Shanghai, China	0.01%	50 µL
Basic fibroblast growth factor	MCE-HY-P70600, Shanghai, China	1×	20 µL
Human Activin A	MCE-HY-P70311, Shanghai, China	1×	25 µL
Pen strep	Gibco-15070063, Shanghai, China	1×	0.5 mL

## Data Availability

Data presented in this study are available upon request from the corresponding author.

## Supplementary Materials

The following supporting information can be downloaded at: https://www.mdpi.com/article/10.3390/ijms26073122/s1.
