# Correction: Liu et al. Chicken Primordial Germ Cells Do Not Proliferate in Insulin-Lacking Media. *Int. J. Mol. Sci.* 2025, *26*, 3122

**DOI:** 10.3390/ijms27062518

**Published:** 2026-03-10

**Authors:** Xin Liu, Jun Wu, Yixiu Peng, Hongwu Qian, Xiaoqian Lv, Fan Li, Kai Jin, Yingjie Niu, Jiuzhou Song, Wei Han, Guohong Chen, Bichun Li, Qisheng Zuo

**Affiliations:** 1 Key Laboratory of Animal Genetics, Breeding and Molecular Design of Jiangsu Province, College of Animal Science and Technology, Yangzhou University, Yangzhou 225009, China; 2 Joint International Research Laboratory of Agriculture and Agri-Product Safety of Ministry of Education of China, Yangzhou University, Yangzhou 225009, China; 3 Animal & Avian Sciences, University of Maryland, College Park, MA 20742, USA; 4 Poultry Institute, Chinese Academy of Agricultural Sciences Poultry Institute of Jiangsu, Yangzhou 225003, China; 5 College of Biotechnology, Jiangsu University of Science and Technology, Zhenjiang 212100, China

In the original publication [1], there was a mistake in Figure 1. There appears to be an unintended shift between the nuclear and cytoplasmic signals in the merged image of Figure 1C. The corrected culture and characterization of chicken primordial germ cells appear below. The authors state that the scientific conclusions are unaffected. This correction was approved by the Academic Editor. The original publication has also been updated.

**Figure 1:**
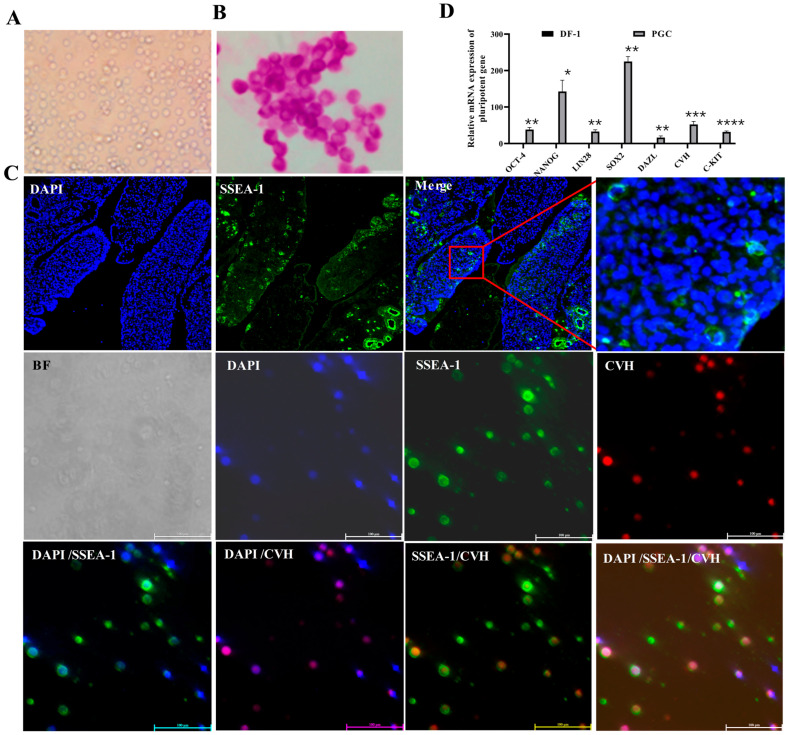
The culture and characterization of chicken primordial germ cells. (**A**) Morphological observation of PGC cells after freezing and thawing; scale bar = 20 μm. (**B**) PAS staining diagram; bar = 10 μm. (**C**) Immunofluorescence of SSEA-1/CVH in PGCs in the gonads and PGCs cultured in vitro; bar = 100 μm. Red square shows a partial enlargement of a specific area in the ‘Merge’ image, used to display details more clearly; red arrow indicates PGCs. (**D**) Expression of pluripotency and germline marker genes; *n* = 3, * *p* < 0.05, ** *p* < 0.01, *** *p* < 0.001, and **** *p* < 0.0001.
