# Supplementary material for: Chicken Primordial Germ Cells Do Not Proliferate in Insulin-Lacking Media
Source: Int J Mol Sci. 2025 Mar 28;26(7):3122. doi: 10.3390/ijms26073122 (PMC11988930; doi:10.3390/ijms26073122)

Figure S1. Representative images of chicken PGCs grown by purification culture at different time points during the process of establishment. PGCs proliferated while other cells died; these included hemocytes, fibroblasts and other cells. Scale bar=20 μm. Black arrows indicate PGCs.

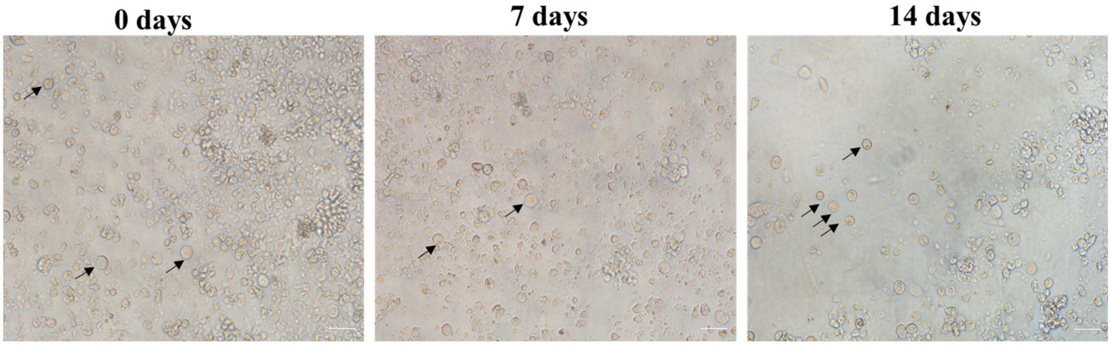

Table S1. Real-time PCR primer sequences.

Function classification	Target gene	Primer sequence (5′ → 3′)	Length	Sources
Cell adhesion	ZO-1	F: TAAGGGGAAGCCAACTGATG<br>R: GAAGGAGCAGGAGGAGGAGT	99bp	XM_046925214.1
	Occludin	CTCCCGGCTGCCATTTTAAG<br>CGTCGTCCACGTAGTAGGAG	151bp	XM_025144247.2
	JAM-A	F: CAGACCCCTACAAGAACCGC<br>R: CACCTGGACGATGAGGTTGA	149bp	NM_001083366.1
	Claudin	ACCAAACAATGCACTGCCAC<br>TCCCTCTTTGGTGAGCTTGG	174bp	NM_001013611.2
	β-catenin	F: ATTTGTGCGCTCCGTCACCT<br>R: ACCTTGTTCACGCAGTGGGG	201bp	NM_205081.3
Pluripotent genes	Lin28B	F: GGCAACATGGCCGAAGCAG<br>R: AAGACATCAACCGGGGACTC	194bp	NM_001034818.2
	OCT-4	F: CGGGATCTCCATGAACAACAG<br>R: CTGGCCCCAGGCAGGTAA	185bp	NM_001309372.2
	NANOG	F: TTGGAAAAGGTGGAACAAGC<br>R: GGTGCTCTGGAAGCTGTAGG	140bp	NM_001146142.2
	SOX2	F: GTGAACCAGAGGATGGACAGTTACG<br>R: TGCGAGCTGGTCATGGAGTTG	185bp	NM_205188.3
PGCs marker genes	CVH	F: GCAACTTCGGTAGCATCA<br>R: TTGGACAGCCATTTCTTC	132bp	XM_046940512.1
	DAZL	F: CTCCTGCACCGCAATTCCAT<br>R: TCTTTGCTCCCCAGGAACCC	209bp	XM_046910203.1
	C-KIT	F: TGCTCGGTTGTAGAATTGCAG<br>R: AGTACACATCGGTAGCATCTGA	78bp	NM_204361.1
Apoptosis	C-MYC	F: ACACAACTACGCTGCTCCTC<br>R: TTCGCCTCTTGTCGTTCTCC	155bp	NM_001030952.2
	Caspase-3	F: CTGAAGGCTCCTGGTTTA<br>R: TGCCACTCTGCGATTTAC	104bp	NM_204725.2
	Caspase-6	F: CCTACACCAACCACCAC<br>R: TCTGCCAAAGTCCCAC	188bp	NM_001396146.1
	BAX	F: TCCATTCAGGTTCTCTTGACC<br>R: GCCAAACATCCAAACACAGA	119bp	XM_040662228.2
	BCL-2	F: CCAAGCAAAAAGAGGAGTCACG<br>R: ACCGTTATACCTAATGCAGCCA	120bp	XM_046910476.1
PI3K-AKT pathway	PI3K	F: CTTCTGGAGTCCTATTGTCG<br>R: CACCTTCTGGGTCTCATCTT	132bp	XM_046923916.1
	AKT	F: GCCGTGAGCCCAGTTAGG<br>R: AGCTACTTATGGCTGCGGGA	153bp	NM_205055.2
	mTOR	F: AACCACTGCTCGCCACAATGC<br>R: CATAGGATCGCCACACGGATTAGC	120bp	XM_417614.6
	COL6A2	F: AGAATTTCCACAGGGCCCAC<br>R: TGATCTGAGCCAGGGCATTG	186bp	NM_205348.4
	AREG	F: CTCAGGAAGGGGCGTGGAG<br>R: CTCCTCGGTTTGCCAGCAT	86bp	XM_015276315.4
	COL6A3	F: AGCCTCCTCCAACAGATCCA<br>R: GTGACCCCAGCGTCATAGAG	215bp	XM_046921233.1
	SGK1	F: ACAAGCAGCCCTATGACA<br>R: TCTCGCAGAGTTGGTAAT	167bp	XM_046912929.1
	ITGA8	F: TACCAGAGACGCTCAGTAACG<br>R: GTGCCTTGTTGTTGCGTTCA	247bp	NM_205288.3
	FGF19	F: GTCCAGACGGCTACAACG<br>R: GTGAGACAGCGGGAGAAA	113bp	NM_204674.3
	CREB3L1	F: GCGCCAGACATTTACACAACT<br>R: TTCATGCGTCCTTGCCAGAT	180bp	XM_046919538.1
	EREG	F: TGGGATAACCAGGTGCAAGC<br>R: GCCTGCGATAGAGATGGCAA	220bp	NM_001001203.2
Wnt pathway	Wnt-5b	CTGGCTGCTTTGTCATCCCT<br>CCTTCTCCTTGCTGCCGATT	191bp	XM_046907576.1
	Wnt-5a	GCTTGGATTACAGCACTGCC<br>CATTGTGCTGCCAAGTGTCC-	104bp	NM_001396502.1
	FGF8	F: CCTTCGCTTAGGGATGTTT<br>R: GTTCAGTTCGGTTGGGTTG	141bp	XM_046942768.1
Internal reference	β-actin	F: CAGCCATCTTTCTTGGGTAT<br>R: CTGTGATCTCCTTCTGCATCC	169bp	NM_205518.1

Figure S2 FPKM of DEGs in PGCs

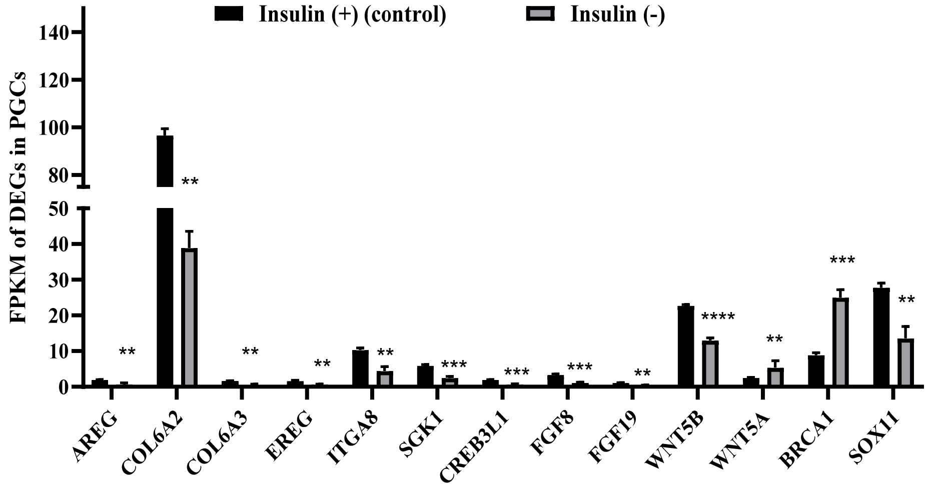

Supplement: Supplementary file 1 [file ijms-26-03122-s001.zip › ijms-3534165-supplementary.pdf]
